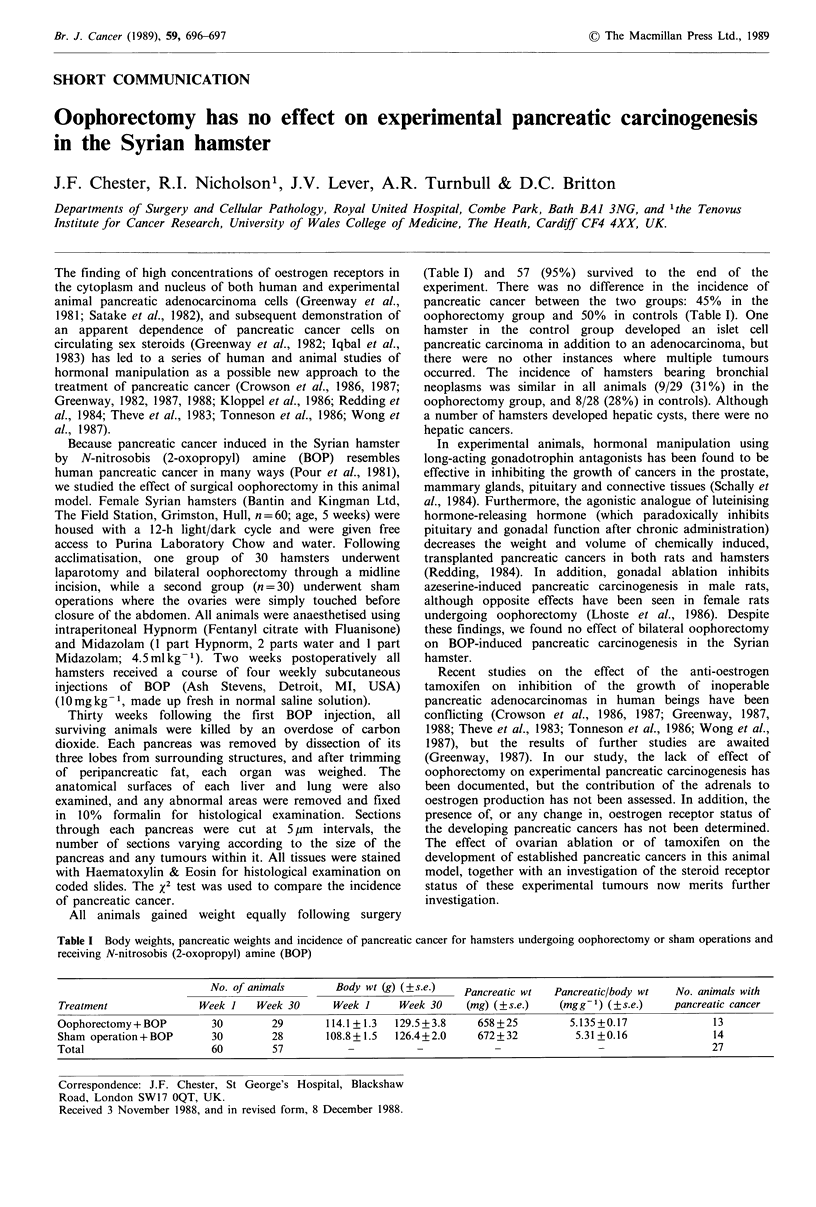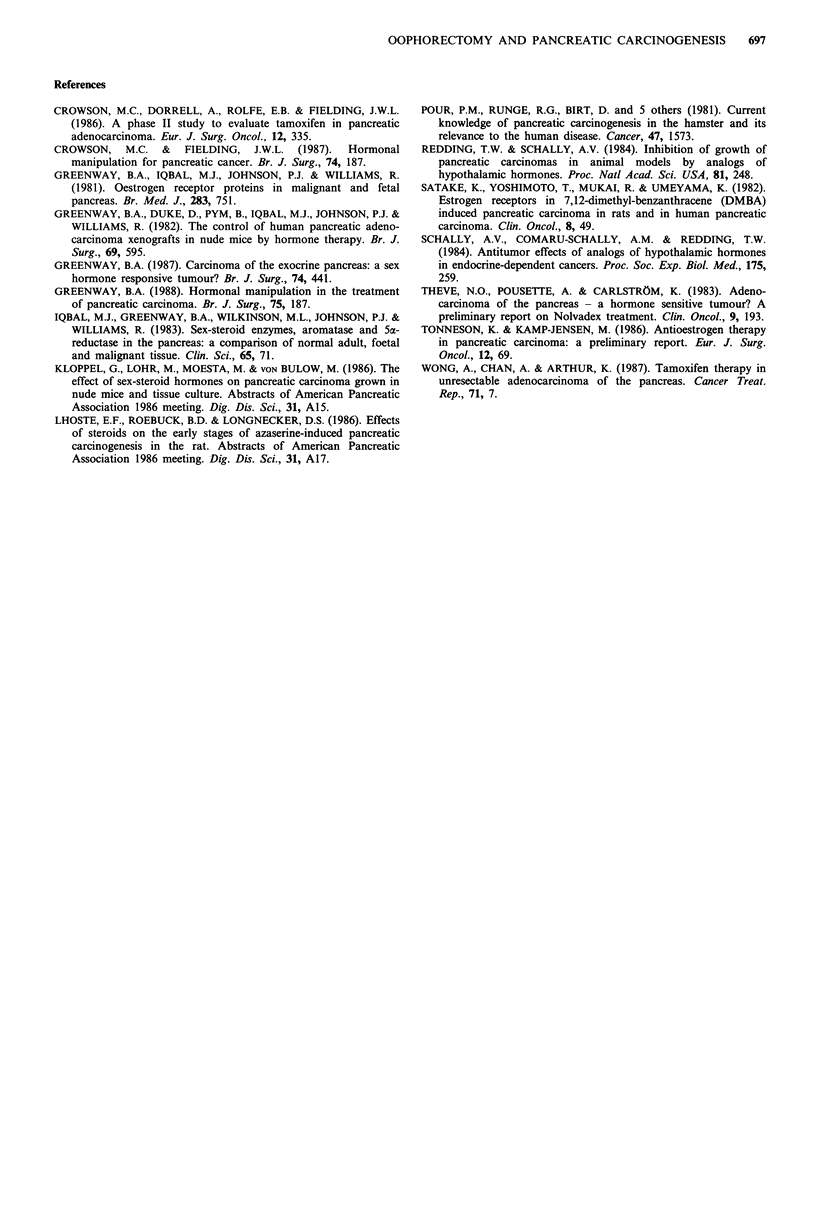# Oophorectomy has no effect on experimental pancreatic carcinogenesis in the Syrian hamster.

**DOI:** 10.1038/bjc.1989.145

**Published:** 1989-05

**Authors:** J. F. Chester, R. I. Nicholson, J. V. Lever, A. R. Turnbull, D. C. Britton

**Affiliations:** Department of Surgery, Royal United Hospital, Combe Park, Bath, UK.


Br. J. Cancer (1989), 59, 696–697 © The Macmillan Press Ltd., 1989

SHORT COMMUNICATION

### Oophorectomy has no effect on experimental pancreatic carcinogenesis in the Syrian hamster

J.F. Chester, R.I. Nicholson[1], J.V. Lever, A.R. Turnbull & D.C. Britton

*Departments of Surgery and Cellular Pathology, Royal United Hospital, Combe Park, Bath BA1 3NG, and [1]the Tenovus Institute for Cancer Research, University of Wales College of Medicine, The Heath, Cardiff CF4 4XX, UK.*

The finding of high concentrations of oestrogen receptors in the cytoplasm and nucleus of both human and experimental animal pancreatic adenocarcinoma cells (Greenway *et al.*, 1981; Satake *et al.*, 1982), and subsequent demonstration of an apparent dependence of pancreatic cancer cells on circulating sex steroids (Greenway *et al.*, 1982; Iqbal *et al.*, 1983) has led to a series of human and animal studies of hormonal manipulation as a possible new approach to the treatment of pancreatic cancer (Crowson *et al.*, 1986, 1987; Greenway, 1982, 1987, 1988; Kloppel *et al.*, 1986; Redding *et al.*, 1984; Theve *et al.*, 1983; Tonneson *et al.*, 1986; Wong *et al.*, 1987).

Because pancreatic cancer induced in the Syrian hamster by *N*-nitrosobis (2-oxopropyl) amine (BOP) resembles human pancreatic cancer in many ways (Pour *et al.*, 1981), we studied the effect of surgical oophorectomy in this animal model. Female Syrian hamsters (Bantin and Kingman Ltd, The Field Station, Grimston, Hull, $n=60$; age, 5 weeks) were housed with a 12-h light/dark cycle and were given free access to Purina Laboratory Chow and water. Following acclimatisation, one group of 30 hamsters underwent laparotomy and bilateral oophorectomy through a midline incision, while a second group ($n=30$) underwent sham operations where the ovaries were simply touched before closure of the abdomen. All animals were anaesthetised using intraperitoneal Hypnorm (Fentanyl citrate with Fluanisone) and Midazolam (1 part Hypnorm, 2 parts water and 1 part Midazolam; $4.5\,ml\,kg^{-1}$). Two weeks postoperatively all hamsters received a course of four weekly subcutaneous injections of BOP (Ash Stevens, Detroit, MI, USA) ($10\,mg\,kg^{-1}$, made up fresh in normal saline solution).

Thirty weeks following the first BOP injection, all surviving animals were killed by an overdose of carbon dioxide. Each pancreas was removed by dissection of its three lobes from surrounding structures, and after trimming of peripancreatic fat, each organ was weighed. The anatomical surfaces of each liver and lung were also examined, and any abnormal areas were removed and fixed in 10% formalin for histological examination. Sections through each pancreas were cut at $5\,\mu m$ intervals, the number of sections varying according to the size of the pancreas and any tumours within it. All tissues were stained with Haematoxylin & Eosin for histological examination on coded slides. The $\chi^2$ test was used to compare the incidence of pancreatic cancer.

All animals gained weight equally following surgery (Table I) and 57 (95%) survived to the end of the experiment. There was no difference in the incidence of pancreatic cancer between the two groups: 45% in the oophorectomy group and 50% in controls (Table I). One hamster in the control group developed an islet cell pancreatic carcinoma in addition to an adenocarcinoma, but there were no other instances where multiple tumours occurred. The incidence of hamsters bearing bronchial neoplasms was similar in all animals (9/29 (31%) in the oophorectomy group, and 8/28 (28%) in controls). Although a number of hamsters developed hepatic cysts, there were no hepatic cancers.

In experimental animals, hormonal manipulation using long-acting gonadotrophin antagonists has been found to be effective in inhibiting the growth of cancers in the prostate, mammary glands, pituitary and connective tissues (Schally *et al.*, 1984). Furthermore, the agonistic analogue of luteinising hormone-releasing hormone (which paradoxically inhibits pituitary and gonadal function after chronic administration) decreases the weight and volume of chemically induced, transplanted pancreatic cancers in both rats and hamsters (Redding, 1984). In addition, gonadal ablation inhibits azeserine-induced pancreatic carcinogenesis in male rats, although opposite effects have been seen in female rats undergoing oophorectomy (Lhoste *et al.*, 1986). Despite these findings, we found no effect of bilateral oophorectomy on BOP-induced pancreatic carcinogenesis in the Syrian hamster.

Recent studies on the effect of the anti-oestrogen tamoxifen on inhibition of the growth of inoperable pancreatic adenocarcinomas in human beings have been conflicting (Crowson *et al.*, 1986, 1987; Greenway, 1987, 1988; Theve *et al.*, 1983; Tonneson *et al.*, 1986; Wong *et al.*, 1987), but the results of further studies are awaited (Greenway, 1987). In our study, the lack of effect of oophorectomy on experimental pancreatic carcinogenesis has been documented, but the contribution of the adrenals to oestrogen production has not been assessed. In addition, the presence of, or any change in, oestrogen receptor status of the developing pancreatic cancers has not been determined. The effect of ovarian ablation or of tamoxifen on the development of established pancreatic cancers in this animal model, together with an investigation of the steroid receptor status of these experimental tumours now merits further investigation.

**Table I** Body weights, pancreatic weights and incidence of pancreatic cancer for hamsters undergoing oophorectomy or sham operations and receiving *N*-nitrosobis (2-oxopropyl) amine (BOP)

| | *No. of animals* | | *Body wt (g) (±s.e.)* | | | | |
|---|---|---|---|---|---|---|---|
| *Treatment* | *Week 1* | *Week 30* | *Week 1* | *Week 30* | *Pancreatic wt (mg) (±s.e.)* | *Pancreatic/body wt ($mg\,g^{-1}$) (±s.e.)* | *No. animals with pancreatic cancer* |
| Oophorectomy+BOP | 30 | 29 | 114.1±1.3 | 129.5±3.8 | 658±25 | 5.135±0.17 | 13 |
| Sham operation+BOP | 30 | 28 | 108.8±1.5 | 126.4±2.0 | 672±32 | 5.31±0.16 | 14 |
| Total | 60 | 57 | – | – | – | – | 27 |

Correspondence: J.F. Chester, St George's Hospital, Blackshaw Road, London SW17 0QT, UK.
Received 3 November 1988, and in revised form, 8 December 1988.

### References

CROWSON, M.C., DORRELL, A., ROLFE, E.B. & FIELDING, J.W.L. (1986). A phase II study to evaluate tamoxifen in pancreatic adenocarcinoma. *Eur. J. Surg. Oncol.*, **12,** 335.

CROWSON, M.C. & FIELDING, J.W.L. (1987). Hormonal manipulation for pancreatic cancer. *Br. J. Surg.*, **74,** 187.

GREENWAY, B.A., IQBAL, M.J., JOHNSON, P.J. & WILLIAMS, R. (1981). Oestrogen receptor proteins in malignant and fetal pancreas. *Br. Med. J.*, **283,** 751.

GREENWAY, B.A., DUKE, D., PYM, B., IQBAL, M.J., JOHNSON, P.J. & WILLIAMS, R. (1982). The control of human pancreatic adenocarcinoma xenografts in nude mice by hormone therapy. *Br. J. Surg.*, **69,** 595.

GREENWAY, B.A. (1987). Carcinoma of the exocrine pancreas: a sex hormone responsive tumour? *Br. J. Surg.*, **74,** 441.

GREENWAY, B.A. (1988). Hormonal manipulation in the treatment of pancreatic carcinoma. *Br. J. Surg.*, **75,** 187.

IQBAL, M.J., GREENWAY, B.A., WILKINSON, M.L., JOHNSON, P.J. & WILLIAMS, R. (1983). Sex-steroid enzymes, aromatase and 5α-reductase in the pancreas: a comparison of normal adult, foetal and malignant tissue. *Clin. Sci.*, **65,** 71.

KLOPPEL, G., LOHR, M., MOESTA, M. & VON BULOW, M. (1986). The effect of sex-steroid hormones on pancreatic carcinoma grown in nude mice and tissue culture. Abstracts of American Pancreatic Association 1986 meeting. *Dig. Dis. Sci.*, **31,** A15.

LHOSTE, E.F., ROEBUCK, B.D. & LONGNECKER, D.S. (1986). Effects of steroids on the early stages of azaserine-induced pancreatic carcinogenesis in the rat. Abstracts of American Pancreatic Association 1986 meeting. *Dig. Dis. Sci.*, **31,** A17.

POUR, P.M., RUNGE, R.G., BIRT, D. and 5 others (1981). Current knowledge of pancreatic carcinogenesis in the hamster and its relevance to the human disease. *Cancer*, **47,** 1573.

REDDING, T.W. & SCHALLY, A.V. (1984). Inhibition of growth of pancreatic carcinomas in animal models by analogs of hypothalamic hormones. *Proc. Natl Acad. Sci. USA*, **81,** 248.

SATAKE, K., YOSHIMOTO, T., MUKAI, R. & UMEYAMA, K. (1982). Estrogen receptors in 7,12-dimethyl-benzanthracene (DMBA) induced pancreatic carcinoma in rats and in human pancreatic carcinoma. *Clin. Oncol.*, **8,** 49.

SCHALLY, A.V., COMARU-SCHALLY, A.M. & REDDING, T.W. (1984). Antitumor effects of analogs of hypothalamic hormones in endocrine-dependent cancers. *Proc. Soc. Exp. Biol. Med.*, **175,** 259.

THEVE, N.O., POUSETTE, A. & CARLSTRÖM, K. (1983). Adenocarcinoma of the pancreas – a hormone sensitive tumour? A preliminary report on Nolvadex treatment. *Clin. Oncol.*, **9,** 193.

TONNESON, K. & KAMP-JENSEN, M. (1986). Antioestrogen therapy in pancreatic carcinoma: a preliminary report. *Eur. J. Surg. Oncol.*, **12,** 69.

WONG, A., CHAN, A. & ARTHUR, K. (1987). Tamoxifen therapy in unresectable adenocarcinoma of the pancreas. *Cancer Treat. Rep.*, **71,** 7.